# Event and Comment

**Published:** 1916-10

**Authors:** 


### DENTAL REGISTER

Vol. LXX OCTOBER, 1916 No. 10

### EVENT AND COMMENT

FOCAL INFECTION AND SYSTEMATIC DISEASE. We are printing in this issue three essays, contributed to the section on Stomatology of the American Medical Association at its recent annual meeting, dealing with the various phases of the systemic diseases produced from pathologic conditions associated with the teeth. This subject has been presented to the dental profession so emphatically and persistently during the past year by the medical profession, and certain dental writers, that many dentists have impatiently resented the suggestion as an unwarranted imputation intended to cover the incapacity of the medical profession to cope with certain systemic disorders. Dentists were at first indignant, then skeptical, and later have become indifferent. The more thoughtful men in the dental profession, however, have taken the sensible position that there may be truth in the idea and are revising methods of practice in accord with all that becomes definitely known, or that seems logically possible to make focal infection from dental diseases impracticable. It is unfortunate that we can not get a unanimity of action by the entire medical and dental professions on so important a matter. Thoughtless dentists are proceeding with careless root canal treatments because they have not been convinced by the statements so far made as to a definite connection between local and systemic diseases, and medical men are insisting that every suspicious tooth shall be removed from the mouth of patients who do not respond promptly to usual medical treatment. Probably much injury has and will result from these antagonistic attitudes while the matter is being worked out to a clearly defined result. It may seem unfortunate that even our scientific workers and some of our most earnest clinicians and writers have taken strongly antagonistic attitudes on this very important question; but we should expect this, and really it will develop a more careful research that will be likely to establish the truth. It is a complicated problem that will take time and much wisdom to solve. The work being done by Hartzell and others on the scientific side, and the clinical application of every scientific fact in treatment by the best men in the profession is bound to give definite and convincing data, if we can only be patient and broad-minded enough to wait; and in the meantime each should contribute what he can by making his practice conform to the generally accepted principles of oral and dental hygiene.

The three papers we are printing in this issue are from reliable, scientific men, and we believe they are most conservative in their statements and deductions. The report of Dr. Hartzell of his findings, as printed in the May issue of the *National Dental Journal* are most convincing and are scientifically accurate. On the other hand a very able paper is published in the September issue of the *Journal of Allied Dental Societies*, by Dr. Percy R. Howe, in which he makes the statement, that "he believes that the theory of localized foci of infection in the mouth as the cause of general systemic conditions, is far from proved." In fact, this writer implies that the position taken by those who believe that diseased dental organs are large contributors to systemic diseases have greatly exaggerated their claims. It is well that we examine so important a matter with the greatest care and precision, and while we do not like the idea of making a contest or court trial of a scientific matter, it may be just as well that the issue be made as strong as possible, even to the controversial, in order that when the decision is reached it will be a definitely established fact. It is a big job, and one of far reaching consequences, and every practitioner of dentistry and medicine should have enough interest in its correct adjustment to at least keep posted as to the results of all scientific researches and such clinical reports as may assist in making methods of practice more conservative.

